# Generalizations from Six Years of Tuberculin

**Published:** 1898-04

**Authors:** James T. Whittaker


### GENERALIZATIONS FROM SIX YEARS OF TUBERCULIN.

BY JAMES T. WHITTAKER, M. D. (*Brit. Med. Jour.*, Oct., 1897 p. 1053).

The use of tuberculin should be studied from the double standpoints of diagnosis and treatment. The value of it in the disclosure of tuberculosis in man was not at once rightly recognized, on account of alleged dangers in the use of it; but in cattle it soon met its proper appreciation, and is, perhaps, nowhere more extensively and satisfactorily used than in the United States. Aside from the objections urged by ignorant and prejudiced people not worthy of notice, four apparently valid arguments accumulated in the course of time against the use of tuberculin in man:

1. That the reaction occurred in the absence of tuberculosis. This has been met by the demonstration of concealed depots.

2. That the reaction occurs in other diseases—syphilis, lepra, actinomycosis, etc. This objection has been met by the demonstration of tuberculosis coincident with or complicating these maladies.

3. That the reaction will occur in the most quiescent case, even where the bacilli are not only concealed, but encapsulated, and depots are partly calcified. This is, of course, no objection at all, but a most striking conformation.

4. That the use of it is dangerous in that it not only awakens the disease from latent sources, but disseminates it over the body, and thus leads to auto-infection and increases the danger of infection of others. This conclusion rests upon a false premise, and confounds the post with the propter hoc.

The best results were attained in office practice; in the first place in the way of diagnosis. Disease treated hitherto without a diagnosis, or for something else, may be cleared up by this method. Thus an obstinate or frequently recurring dyspepsia, an amenorrhœa, a neurasthenia, an anæmia, a rheumatism, an adenitis, a little low fever (to say nothing of acute cases of chills, night sweats, catarrh, and coughs) may have their true nature revealed in this way. These are the cases reported (aside from chills and fever) in which the treatment furnishes the best results. Tuberculin gives good results in childhood; they react more favorably to tuberculin than adults, so that the so-called apex catarrh of childhood will often clear up within three weeks.

For diagnostic purpose the first dose in cases of suspected light infection should be 5 mg., the second 10 mg. or 1 cg., the third 2 cg. These doses may be given on succeeding days, or, better, every other day, preferably in the evening, that the temperature records may be properly observed on the following day. Failure to get reaction from three tests exclude concealed tuberculosis. The crude tuberculin diluted one hundred times, preferably with one-half per cent. solution of carbolic acid, makes, of course, a one per cent. solution, of which one interspace of the Koch syringe represents 1 cg. One interspace of this one per cent. solution diluted ten times makes 1 mg. For therapeutic use begin with one-tenth of a milligramme.

The highest value of tuberculin is the diagnostic value, which is supreme, and which enables us to distinguish the disease from the start as a tuberculosis, before the development of sepsis or other complications which go to make up phthisis.—*New York Postgraduate.*

